# Spiramycin-Metronidazole Synergism Testing on Aggregatibacter actinomycetemcomitans

**DOI:** 10.7759/cureus.90587

**Published:** 2025-08-20

**Authors:** Thomas E Rams, Joanie Faucher

**Affiliations:** 1 Department of Periodontology and Oral Implantology, Temple University Maurice H. Kornberg School of Dentistry, Philadelphia, USA

**Keywords:** aggregatibacter actinomycetemcomitans, amoxicillin, ciprofloxacin, metronidazole, periodontal, periodontitis, spiramycin, subgingival microbiota, synergism

## Abstract

Background

The potential synergism of spiramycin-metronidazole in combination against *Aggregatibacter actinomycetemcomitans*, a major pathogen in human periodontitis, peri-implantitis, and some non-oral infections, was studied in vitro in comparison to combinations of amoxicillin-metronidazole and ciprofloxacin-metronidazole.

Methods

Minimal inhibitory concentrations (MICs) for spiramycin, amoxicillin, ciprofloxacin, and metronidazole were determined individually against four periodontal *A. actinomycetemcomitans *clinical isolates. Synergism testing for the combinations of spiramycin-metronidazole, amoxicillin-metronidazole, and ciprofloxacin-metronidazole was performed with gradient diffusion strips for each antibiotic pair placed onto *A. actinomycetemcomitans*-inoculated *Haemophilus *test medium in a cross formation at the intersection of each of their individual MICs against *A. actinomycetemcomitans.*Fractional inhibitory concentration index values assessed the antibiotic interactions.

Results

Spiramycin and metronidazole individually had poor antimicrobial activity against *A. actinomycetemcomitans*. However, lower MIC values and less *A. actinomycetemcomitans *resistance were found for both antibiotics when tested in combination. Spiramycin-metronidazole synergism was additionally detected against one *A. actinomycetemcomitans *clinical isolate. In comparison, combinations of amoxicillin-metronidazole and ciprofloxacin-metronidazole exhibited antimicrobial synergism against all four *A. actinomycetemcomitans *strains.

Conclusions

Spiramycin and metronidazole exerted greater in vitro antimicrobial activity in combination than individually against *A. actinomycetemcomitans*. Antimicrobial synergism between spiramycin and metronidazole was also found with one *A. actinomycetemcomitans *clinical isolate, representing the first detection of spiramycin-metronidazole synergism against *A. actinomycetemcomitans*. However, antimicrobial synergism against *A. actinomycetemcomitans *was less frequently detected with spiramycin-metronidazole as compared to combinations of amoxicillin-metronidazole or ciprofloxacin-metronidazole. These findings may help clinicians in the selection of effective antimicrobial therapies against oral and non-oral infections involving *A. actinomycetemcomitans*.

## Introduction

*Aggregatibacter* (formerly *Actinobacillus*) *actinomycetemcomitans* is a fastidious, capnophilic, facultatively anaerobic, gram-negative, non-motile coccobacillus whose primary habitat is in the human oral cavity, colonizing mucosal surfaces and tooth biofilms [1]. The species is a key pathogen in molar-incisor (aggressive) periodontitis in adolescents and young adults [2], severe periodontitis in older adults [3], and infectious forms of dental implant failure (peri-implantitis) [4]. *A.* *actinomycetemcomitans* is also occasionally the cause of serious non-oral infections, including infective endocarditis, brain abscesses, endophthalmitis and lung infections [5,6].

Combination antibiotic therapy may exert enhanced antimicrobial activity against specific bacterial pathogens, such as *A*. *actinomycetemcomitans*, via drug synergism, where the antimicrobial effects of two antibiotics are greater in combination than their sum individually [7]. Combinations of amoxicillin-metronidazole and ciprofloxacin-metronidazole both exhibit antimicrobial synergism in vitro against strains of *A*. *actinomycetemcomitans* [8-10]. These in vitro findings were supported by clinical outcomes, where in patients with *A*. *actinomycetemcomitans*-associated periodontitis, amoxicillin-metronidazole and ciprofloxacin-metronidazole therapies eradicated *A*. *actinomycetemcomitans* from subgingival biofilms and surrounding soft tissues, and improved clinical periodontal parameters, better than single antibiotic regimens or mechanical/surgical debridement alone [11,12]. Amoxicillin-metronidazole combination therapy also resolved an *A*. *actinomycetemcomitans* infection of oral origin on a heart pacemaker wire, inducing recurrent episodes of septicemia in a patient [13].

Spiramycin plus metronidazole is another antibiotic combination used successfully in the treatment of periodontitis and odontogenic infections [14-17], largely in Europe (especially France), Canada, Central and South America, Vietnam and the Middle East, but not in the United States, where spiramycin is available only by special permission from United States Food and Drug Administration for treatment of toxoplasmosis in pregnant women. The combination of spiramycin-metronidazole has a broad complementary spectrum of antimicrobial activity, with spiramycin most active against gram-positive bacteria, and metronidazole against anaerobic bacteria and protozoa [18]. Decreases in subgingival *A*. *actinomycetemcomitans* prevalence and levels occurred after systemic spiramycin-metronidazole therapy in periodontitis patients [15,17] despite in vitro resistance of *A*. *actinomycetemcomitans* to both spiramycin and metronidazole individually [15]. This enhanced anti-*A*. *actinomycetemcomitans* effect is suggestive of synergism between the two antibiotics when given in combination against *A*. *actinomycetemcomitans*. However, synergism testing of spiramycin-metronidazole against *A*. *actinomycetemcomitans* is limited so far to three *A*. *actinomycetemcomitans* laboratory reference strains, where no antimicrobial synergism has been detected [18,19]. Because laboratory-adapted reference strains of a microbial species may differ from wild-type clinical isolates of the organism in their antibiotic susceptibility [20], there is a need to further examine the potential of spiramycin-metronidazole synergism against *A*. *actinomycetemcomitans* using wild-type clinical isolates.

To help address this clinically relevant issue, wild-type *A*. *actinomycetemcomitans* clinical isolates were obtained from human periodontitis lesions and used to determine the extent to which spiramycin-metronidazole exerts antimicrobial synergism in vitro against *A*. *actinomycetemcomitans* in comparison to combinations of amoxicillin-metronidazole and ciprofloxacin-metronidazole.

## Materials and methods

A cross-sectional laboratory study of the antimicrobial activity in vitro of selected antibiotics individually and in combination against *A*. *actinomycetemcomitans* clinical isolates was carried out at the Oral Microbiology Testing Service (OMTS) Laboratory at Temple University School of Dentistry, Philadelphia, United States. The OMTS Laboratory has been licensed for high complexity bacteriologic analysis and bacterial susceptibility testing by the Pennsylvania Department of Health and certified by the United States Centers for Medicare and Medicaid Services to be in compliance with Clinical Laboratory Improvement Amendments (CLIA) standards required of clinical laboratories engaged in diagnostic testing of human specimens in the United States.

The Temple University Office for Human Subjects Protections Institutional Review Board classified this study (IRB protocol no. 13442) as exempt since it involved secondary use of pre-existing clinical isolates anonymized with removal of unique patient identifiers and the absence of any human subject-investigator contact or interaction.

Bacterial strains

Four wild-type *A*. *actinomycetemcomitans* clinical isolates were recovered on pre-reduced trypticase soy-bacitracin-vancomycin (TSBV) agar, a selective medium for *A*. *actinomycetemcomitans* [21], from subgingival biofilms cultivated at the OMTS Laboratory from four adults with severe periodontitis. The subgingival biofilm specimens were obtained and submitted to the laboratory, as previously described [22], by practicing periodon­tists seeking microbiological analysis and in vitro antibiotic susceptibility testing for periodontal patient treatment planning. *A. actinomycetemcomitans* was identified on TSBV culture medium, after incubation at 35ºC for three days in 5% CO_2_-95% air, as forming catalase-positive, circular, convex, translucent, adherent, glistening colonies with slightly irregular edges and an inner star-shaped structure [21].

Antibiotic susceptibility testing

Susceptibility testing in vitro of spiramycin, amoxicillin, ciprofloxacin, and metronidazole individually against each of the four *A*. *actinomycetemcomitans* clinical isolates followed methods previously described [22]. In brief, direct colony suspensions of pure *A*. *actinomycetemcomitans* from TSBV medium plates were prepared and adjusted to a 1.0 McFarland turbidity standard, providing approximately 3 x 10^8^ organisms/ml. Using a sterile cotton-tip swab, the four isolate suspensions were applied separately to 150-mm diameter culture plates containing *Haemophilus* test medium (Becton, Dickinson and Company, New Jersey, United States). After drying, antibiotic-impregnated gradient diffusion strips for spiramycin, amoxicillin, ciprofloxacin and metronidazole (MIC Test Strip, Liofilchem s.r.l., Roseto degli Abruzzi, Italy), with predefined antibiotic gradients immobilized across 15 two-fold dilutions on one side, and a minimal inhibitory concentration (MIC) interpretive scale printed on the other side, were placed in duplicate onto the inoculated *Haemophilus* test medium surfaces. After 24 hours of incubation at 35ºC in 5% CO_2_-95% air, the intersection between the border of *A*. *actinomycetemcomitans* growth and the strip drug scale was read to determine the MIC in mg/L, following the manufacturer’s instructions.

Since no internationally recognized antibiotic MIC interpretative guidelines specific to *A*. *actinomycetemcomitans* were available, the present study employed resistance breakpoint concentrations of 4 mg/L for spiramycin and 16 mg/L for metronidazole, as previously used with periodontal *A*. *actinomycetemcomitans* by Madinier et al. [23], and 2 mg/L for amoxicillin and 0.03 mg/L for ciprofloxacin, as established by the European Committee on Antimicrobial Susceptibility Testing (EUCAST) [24] for *Haemophilus* species, which are phenotypically similar to *A*. *actinomycetemcomitans* [1]. *A.* *actinomycetemcomitans* isolates with MICs greater than resistance breakpoint concentrations were identified as resistant to the antibiotic or otherwise considered susceptible. 

Antibiotic synergism testing

**Figure 1:**
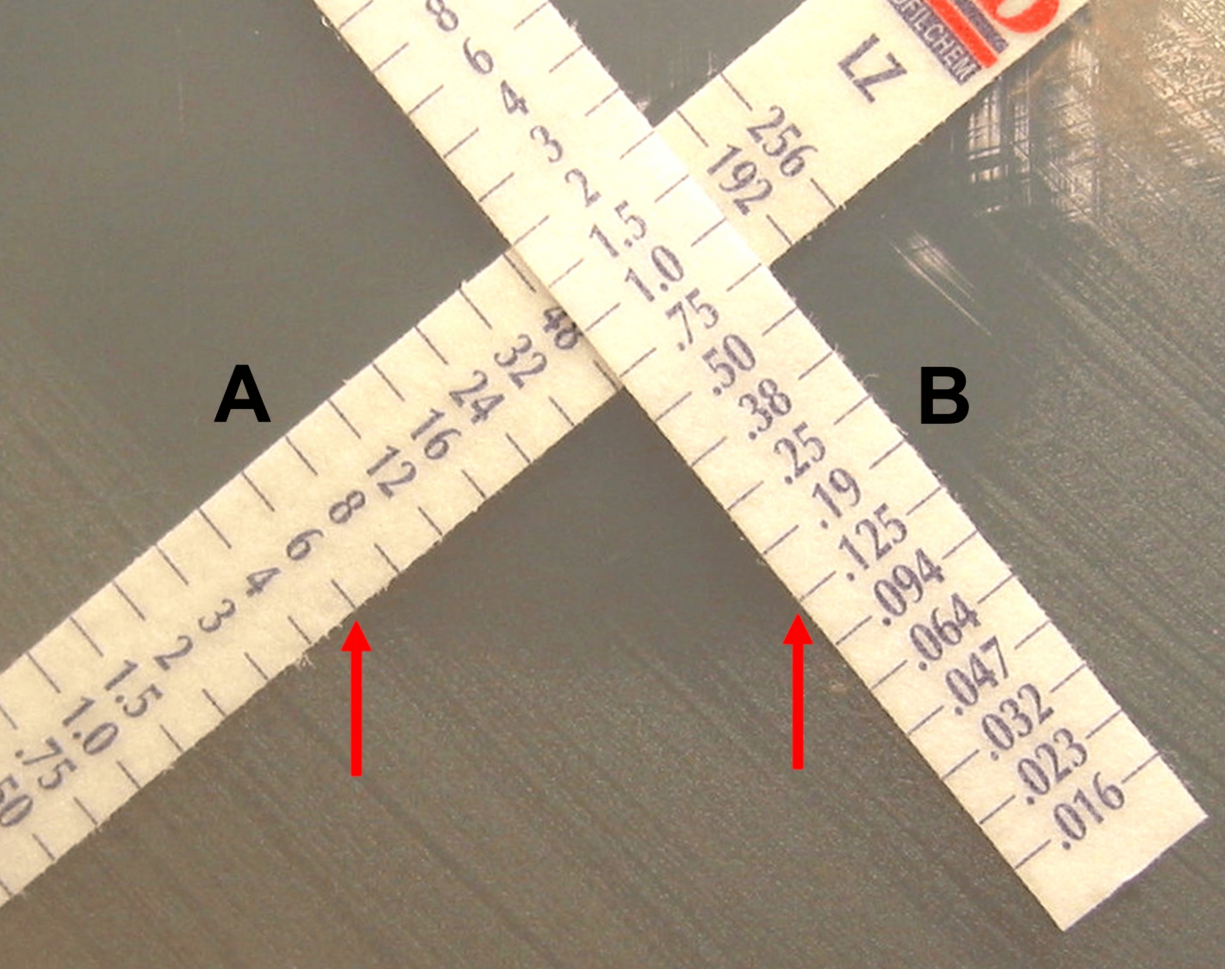
Example of antibiotic gradient diffusion strip placement for in vitro synergism testing Strips for metronidazole (A) and amoxicillin (B) are placed in a cross formation at the intersection of their MIC in individual drug testing against *Aggregatibacter* *actinomycetemcomitans* (48 mg/L for metronidazole and 0.75 mg/L for amoxicillin). MIC for each antibiotic in synergism testing was read, where *A. actinomycetemcomitans* inhibition ellipses intersected each gradient diffusion strip (red arrows) (6 mg/L for metronidazole and 0.125 mg/L for amoxicillin). MIC: minimal inhibitory concentration

Synergism testing of combinations of spiramycin-metronidazole, amoxicillin-metronidazole, and ciprofloxacin-metronidazole on the four *A*. *actinomycetemcomitans* clinical isolates was performed as previously described [25]. Using a vacuum applicator pen (Nema C88; bioMérieux SA, Marcy-l'Étoile, France), gradient diffusion strips for each of the antibiotic combination pairs were placed in duplicate onto *A*. *actinomycetemcomitans*-inoculated *Haemophilus* test medium in a cross formation creating a 90° angle between the two antibiotic strips at the point where their MIC values from mono-drug testing against *A*. *actinomycetemcomitans* intersected on each strip’s MIC interpretive scale. After 48 hours of incubation at 35ºC in 5% CO_2_-95% air, the MIC for each antibiotic was read in mg/L where bacterial inhibition ellipses intersected each gradient diffusion strip (Figure 1). 

For the three drug combinations, fractional inhibitory concentration index (FICI) values were calculated by dividing the MIC of each antibiotic in combination testing against *A*. *actinomycetemcomitans* by the MIC of the antibiotic alone against *A*. *actinomycetemcomitans* and summing the results [25]. FICI values were interpreted as signifying either synergism (FICI ≤0.5), no interaction (FICI >0.5 and ≤4), or antagonism (FICI >4) between antibiotics tested in combination against *A*. *actinomycetemcomitans* [25].

## Results

Antibiotic susceptibility testing

Table 1 presents MIC values for the four antibiotics tested against four *A*. *actinomycetemcomitans* clinical isolates. 

**Table 1:** Minimal inhibitory concentrations (MIC) of antibiotics tested individually against A. actinomycetemcomitans and drug resistance of the clinical isolates ^a^Antibiotic-resistant, MIC exceeds resistance breakpoint concentration of either 4 mg/L for spiramycin, 2 mg/L for amoxicillin, 0.03 mg/L for ciprofloxacin, or 16 mg/L for metronidazole.

Antibiotic	MIC (mg/L) for A. actinomycetemcomitans clinical isolate			
	Isolate #1	Isolate #2	Isolate #3	Isolate #4
spiramycin	>32^a^	>32^a^	>32^a^	>32^a^
amoxicillin	1	0.75	1.5	1
ciprofloxacin	0.016	0.023	0.064^a^	0.064^a^
metronidazole	8	48^a^	24^a^	48^a^

All *A*. *actinomycetemcomitans* clinical isolates were resistant in vitro to spiramycin with MIC values of >32 mg/L. Similarly, three of four *A*. *actinomycetemcomitans* isolates were resistant to metronidazole, with MIC ranging between 24 to 48 mg/L. In comparison, all *A*. *actinomycetemcomitans* strains were susceptible to amoxicillin with MIC ≤1.5 mg/L, and two of four isolates were susceptible to ciprofloxacin (Table 1).

Antibiotic synergism testing

Table 2 reveals the outcome of synergism testing of spiramycin-metronidazole, amoxicillin-metronidazole, and ciprofloxacin-metronidazole against four *A*. *actinomycetemcomitans* clinical isolates.

**Table 2:** Minimal inhibitory concentrations (MIC), fractional inhibitory concentration index (FICI) values, and antimicrobial synergism in vitro of three antibiotic combinations against A. actinomycetemcomitans ^a^Antibiotic-resistant, MIC exceeds resistance breakpoint concentration of either 4 mg/L for spiramycin, 2 mg/L for amoxicillin, 0.03 mg/L for ciprofloxacin, or 16 mg/L for metronidazole. ^b^MIC lower in antibiotic combination vs. individual drug testing. + present; - absent

Antibiotic Combination	*Aggregatibacter* *actinomycetemcomitans* clinical isolate			
	Isolate #1	Isolate #2	Isolate #3	Isolate #4
spiramycin-metronidazole:				
spiramycin MIC (mg/L)	>32^a^	3^b^	12^ab^	16^ab^
metronidazole MIC (mg/L)	8	16^b^	12^b^	24^ab^
FICI	2	0.427	0.875	1
antimicrobial synergism	-	+	-	-
amoxicillin-metronidazole:				
amoxicillin MIC (mg/L)	0.19^b^	0.125^b^	0.25^b^	0.047^b^
metronidazole MIC (mg/L)	1^b^	6^b^	4^b^	3^b^
FICI	0.315	0.292	0.334	0.110
antimicrobial synergism	+	+	+	+
ciprofloxacin-metronidazole:				
ciprofloxacin MIC (mg/L)	0.002^b^	0.004^b^	0.012^b^	0.004^b^
metronidazole MIC (mg/L)	0.5^b^	6^b^	4^b^	2^b^
FICI	0.188	0.299	0.355	0.105
antimicrobial synergism	+	+	+	+

In spiramycin-metronidazole testing, lower MICs were found for both spiramycin and metronidazole when tested in combination against three *A*. *actinomycetemcomitans* isolates as compared to individually. Spiramycin MIC in the presence of metronidazole ranged from 3-16 mg/L against three *A*. *actinomycetemcomitans* strains (Table 2), in comparison to >32 mg/L found with spiramycin alone (Table 1). One *A*. *actinomycetemcomitans* isolate resistant to spiramycin alone was susceptible to the drug in the presence of metronidazole. Lower metronidazole MICs and less *A*. *actinomycetemcomitans* resistance to metronidazole were also found in spiramycin-metronidazole combination testing. With three *A*. *actinomycetemcomitans* isolates, metronidazole MIC ranged from 12 mg/L to 24 mg/L in the presence of spiramycin (Table 2), as compared to 24-48 mg/L when tested alone. The three isolates, which were metronidazole-resistant in mono-drug testing, were susceptible to metronidazole when combined with spiramycin (Table 2). 

A FICI value of 0.427, indicative of antimicrobial synergism, was found for spiramycin-metronidazole against one *A*. *actinomycetemcomitans* isolate. Three other *A*. *actinomycetemcomitans* strains yielded FICI values between 0.875 and 2, indicating no interactions between spiramycin and metronidazole (Table 2).

In both amoxicillin-metronidazole and ciprofloxacin-metronidazole testing, MICs for amoxicillin, ciprofloxacin, and metronidazole were all lower when tested in combination against the *A*. *actinomycetemcomitans* isolates than when tested alone (Table 2). Moreover, none of the *A*. *actinomycetemcomitans* strains were resistant to amoxicillin, ciprofloxacin, or metronidazole in combination testing (Table 2). FICI values of ≤0.5, denoting antimicrobial synergism, were found for both amoxicillin-metronidazole and ciprofloxacin-metronidazole against all four *A*. *actinomycetemcomitans* isolates (Table 2).

No antagonistic interactions were found for any of the tested antibiotic combinations.

## Discussion

The combination of spiramycin-metronidazole revealed greater antimicrobial activity in vitro against three of four wild-type *A*. *actinomycetemcomitans* clinical isolates, resulting in lower MIC values for both drugs and less *A*. *actinomycetemcomitans* in vitro resistance than was achieved individually by the two antibiotics. Spiramycin-metronidazole also exerted antimicrobial synergism against one *A*. *actinomycetemcomitans* strain. However, no synergism occurred between spiramycin and metronidazole with three other wild-type *A*. *actinomycetemcomitans* clinical isolates in the present study, as well as with three previously studied *A*. *actinomycetemcomitans* laboratory reference strains [18,19]. This suggests an estimated effective synergism rate [7] of 14.3% for spiramycin-metronidazole against *A*. *actinomycetemcomitans* (one of seven strains where antimicrobial synergism between the antibiotics was detected). 

Spiramycin-metronidazole synergism was previously identified mostly with obligate anaerobic bacteria, including *Bacteroides* *fragilis*, *Bacteroides* *melaninogenicus* group organisms (presently *Prevotella* and *Porphyromonas* species), *Eubacterium* species, oral anaerobic cocci, and *Propionibacterium* species [7,18,26,27], rather than facultative anaerobic species like *A*. *actinomycetemcomitans*. Among the largely anaerobic bacterial pathogens inhabiting human periodontal pockets, only one strain each of the facultative anaerobic species, *Streptococcus* *constellatus* and *Streptococcus* *intermedius*, in one (2.7%) of 37 untreated severe periodontitis patients was resistant in vitro to both spiramycin and metronidazole [28]. The synergism of spiramycin-metronidazole against *A*. *actinomycetemcomitans*, at a low prevalence in the present study, is the first detection of this antibiotic interaction against a strain of *A*. *actinomycetemcomitans*.

The present study also confirms and expands findings from previous in vitro studies of amoxicillin-metronidazole and ciprofloxacin-metronidazole synergism against *A*. *actinomycetemcomitans *[8-10]. Amoxicillin-metronidazole was synergistic against all four tested *A*. *actinomycetemcomitans* clinical isolates in the present study, in agreement with previous observations of amoxicillin-metronidazole synergism against all 10 *A*. *actinomycetemcomitans* strains in one study [8], and two of eight *A*. *actinomycetemcomitans* strains in another study [10]. Ciprofloxacin-metronidazole was also synergistic against all four *A*. *actinomycetemcomitans* clinical isolates in the present study, as well as with all five *A*. *actinomycetemcomitans* strains in a previous study [9]. These data collectively yield estimated effective synergism rates of 72.7% and 100%, respectively, for amoxicillin-metronidazole and ciprofloxacin-metronidazole against *A*. *actinomycetemcomitans*. The highly effective synergism rates for amoxicillin-metronidazole and ciprofloxacin-metronidazole against *A*. *actinomycetemcomitans* suggest that better treatment responses are more likely with either drug combination than may be expected with spiramycin-metronidazole, with only an estimated 14.3% effective synergism rate. 

The lower MIC values and drug synergism by the study antibiotics in combination against *A*. *actinomycetemcomitans*, as compared to their individual antimicrobial activity (Table 2), suggests that lower combined doses of the antibiotics may be clinically administered when they are given together which retains or improves their therapeutic effectiveness, and reduces the risk of adverse drug side effects/toxicity, in the treatment of patients with *A*. *actinomycetemcomitans* infections. 

The present study has several limitations to be considered. Only a small number of wild-type *A*. *actinomycetemcomitans* clinical isolates of United States origin were studied, which reduces the generalizability of the findings. The synergism testing was performed with culture plate incubation in a microaerophilic atmosphere, similar to Madinier et al. [23], which enhanced the growth of *A*. *actinomycetemcomitans* [29] but reduced the efficacy of metronidazole [30]. The hydroxymetabolite of metronidazole, which is produced in the liver after drug absorption and inhibitory to *A*. *actinomycetemcomitans* [8], was not available on gradient diffusion strips for use in the present study. Mechanisms underlying the observed antibiotic synergisms were not studied and remain to be delineated. Finally, since in vitro and in vivo findings do not necessarily concur, clinical research studies are needed to determine whether synergistic effects of spiramycin-metronidazole are found in vivo and significantly enhance elimination of *A*. *actinomycetemcomitans* infections.

## Conclusions

Spiramycin and metronidazole produced greater antimicrobial activity, with lower MIC values and less *A*. *actinomycetemcomitans* in vitro resistance, when employed together in combination than individually against *A*. *actinomycetemcomitans* clinical isolates. Additionally, the first detection of spiramycin-metronidazole synergism in vitro against *A*. *actinomycetemcomitans* was found with one clinical isolate. However, antimicrobial synergism against *A*. *actinomycetemcomitans* was less frequently detected with spiramycin-metronidazole as compared to combinations of amoxicillin-metronidazole or ciprofloxacin-metronidazole. These findings may help clinicians in the selection of effective antimicrobial therapies against oral and non-oral infections involving *A*. *actinomycetemcomitans*.
